# Influence of Iron on Bone Homeostasis

**DOI:** 10.3390/ph11040107

**Published:** 2018-10-18

**Authors:** Enikő Balogh, György Paragh, Viktória Jeney

**Affiliations:** 1 Research Centre for Molecular Medicine, Faculty of Medicine, University of Debrecen, 4012 Debrecen, Hungary; balogh.eniko@belklinika.com; 2 Department of Internal Medicine, Faculty of Medicine, University of Debrecen, 4012 Debrecen, Hungary; paragh@internal.med.unideb.hu

**Keywords:** bone homeostasis, iron overload, iron deficiency, osteoclast, osteoblast, osteoporosis

## Abstract

Bone homeostasis is a complex process, wherein osteoclasts resorb bone and osteoblasts produce new bone tissue. For the maintenance of skeletal integrity, this sequence has to be tightly regulated and orchestrated. Iron overload as well as iron deficiency disrupt the delicate balance between bone destruction and production, via influencing osteoclast and osteoblast differentiation as well as activity. Iron overload as well as iron deficiency are accompanied by weakened bones, suggesting that balanced bone homeostasis requires optimal—not too low, not too high—iron levels. The goal of this review is to summarize our current knowledge about how imbalanced iron influence skeletal health. Better understanding of this complex process may help the development of novel therapeutic approaches to deal with the pathologic effects of altered iron levels on bone.

## 1. Introduction

Bone is a metabolically active tissue that is continuously being remodeled, which enables growth in childhood, as well as repair and adaptation of the skeleton in adults. During bone remodeling, the adult skeleton is renewed approximately once every ten years. The two major cell types involved in bone remodeling are the osteoclasts, with a function of resorption of bone tissue and osteoblasts, with a role of new bone tissue formation. Osteoclasts originate from the monocyte/macrophage hematopoietic lineage, whereas osteoblasts originate from multipotent mesenchymal stem cells. Differentiation and activity of these two cell types must be tightly orchestrated in order to preserve skeletal health and integrity throughout life.

Iron overload as well as iron deficiency are associated with weakened bones, suggesting that balanced bone homeostasis requires optimal iron levels. Accumulating evidence suggests that both high iron and low iron influence the differentiation and activity of osteoclast and osteoblasts in a way that it promotes bone loss.

## 2. Bone Homeostasis

Bones are often stereotyped as simply a scaffold that holds the body together. But besides supporting the body structurally, bones have many different functions [1]. For example, bones protect our internal organs as well as the central nervous system from injury, and enable us to move. Also, bones serve as a reservoir for phosphorus and calcium and they provide an environment for hematopoiesis that occurs in the red bone marrow. Additionally, bones store energy in a form of lipids in adipose cells present in the yellow marrow.

Despite the first impression, bone is a metabolically active tissue that is continuously being remodeled, which is a process not only important for bone health and homeostasis of phosphorus and calcium, but also because it allows for adaptation of the skeleton to meet changing mechanical needs [2]. In the cycle of bone remodeling, old bone tissue is digested by osteoclasts, and the new bone tissue is made by osteoblasts in a tightly coordinated manner to assure similar rates of resorption and formation thus sustaining skeletal integrity [2]. Bone remodeling is orchestrated by systemic regulators including hormones (e.g., parathyroid-, growth-, and sex hormones), glucocorticoids, prostaglandins, calcitriol, calcitonin, bone morphogenetic proteins (BMP), as well as local regulators including many cytokines and growth factors [1].

### 2.1. Osteoclasts

Osteoclasts, the unique cells involved in bone resorption, originate from myeloid cells of the monocyte/macrophage lineage. Osteoclastogenesis is a multistep process, in which first osteoclast precursors differentiate into mononuclear pre-osteoclast, which then fuse into multinucleated mature osteoclasts, on further differentiation [3]. Terminally differentiated osteoclasts are involved in the resorption of bone tissue. They move on bone surfaces, seal the resorption bays, pump hydrogen ions and secrete proteolytic enzymes into the resorption cavity, to dissolve the inorganic bone matrix and degrade bone matrix proteins [4,5]. Mature osteoclasts are short-lived cells and undergo programmed cell death within a few days following maturation.

Osteoclastogenesis is a tightly regulated process in which diverse cytokines, steroids and lipids play pivotal roles [4,6]. Among them, both macrophage colony-stimulating factor (M-CSF) and receptor activator of nuclear factor κB ligand (RANKL) seem to be necessary to osteoclast differentiation. This notion is sustained by the observations that the absence of a functional M-CSF in the op/op mouse as well as RANKL deficiency are associated with a complete lack of mature osteoclasts resulting in profound osteopetrosis [3,7,8,9,10]. The binding of RANKL to RANK triggers the recruitment of adaptor molecules, such as tumor necrosis factor receptor-associated factor 6 (TRAF6) and eventually leads to the activation of multiple downstream signal transduction pathways, including c-Jun N-terminal kinase (JNK), p38, and extracellular signal-regulated kinase (ERK) pathways, nuclear factor-κB (NF-κB), Src and Akt [11]. Importantly, RANKL/RANK signaling activates different transcription factors such as NF-κB, microphthalmia transcription factor (MITF), c-Fos, and nuclear factor-activated T cells c1 (NFATc1) [11]. Among them, NFATc1 is considered to be the master transcription factor that drives terminal differentiation of osteclasts via regulating numerous osteoclast-specific genes such as tartrate-resistant acid phosphatase (TRAP), cathepsin K and calcitonin receptor [12,13].

Excessive osteoclastogenesis and bone resorption is prevented by osteoprotegerin (OPG) that regulates RANK/RANKL signaling. OPG is a soluble decoy receptor that binds RANKL and thus prevent its binding to RANK, therefore the RANKL-OPG ratio is an important regulator of bone mass and skeletal integrity [8,14].

### 2.2. Osteoblasts

Osteoblasts, which are the unique bone forming cells, originate from multipotent mesenchymal stem cells (MSCs). To accomplish their role, MSCs first need to migrate to the site of active bone resorption, proliferate and differentiate into active osteoblasts. Migration of MSCs is regulated by growth factors deposited within the bone matrix and liberated during bone resorption, such as transforming growth factor beta 1 (TGF-β1), platelet derived growth factor (PDGF), BMP2 and BMP4 [15,16]. Additionally, the activated osteoclast-derived chemokine sphingosine-1-phosphate also plays a critical role in the recruitment of MSCs to the bone resorption cavity [17]. These mechanisms ensure precise coupling of bone resorption and subsequent bone formation and therefore, contribute to maintenance of skeletal integrity [16].

MSCs are able to change into various cell types including osteoblasts, chondrocytes and adipocytes. The processes of commitment and differentiation of MSCs are driven by lineage-specific master transcription factors. Osteogenic differentiation of MSCs is driven by runt-related transcription factor 2 (Runx2), the master osteogenic transcription factor. Importantly, Runx2 deficient mice die shortly after birth because of impaired bone formation due to the absence of differentiated osteoblasts [18,19]. Runx2 has a wide variety of targets that includes the key bone tissue specific proteins such as osteopontin, osteocalcin (OCN), bone sialoprotein (BSP) and alkaline phosphatase (ALP) [20].

Runx2 activation and subsequent osteogenic differentiation of MSCs is triggered by diverse secreted differentiation factors, including TGF-β1, fibroblast growth factor, and upstream signaling pathways such as BMP, Wnt and hedgehog [21,22]. Additionally, recent evidence highlighted the key role of reactive oxygen species (ROS) in osteogenesis as a common regulator of the diverse osteogenic signaling pathways [23]. Results showed that rigorously balanced ROS levels are crucial in proper osteogenic differentiation of MSCs [23].

## 3. Bone Homeostasis in Iron Overload

### 3.1. Iron Overload

Iron is a Janus face element, being both essential for life and dangerous, due to its involvement in unfettered ROS production. Because of this, diverse proteins keep the uptake (e.g., divalent metal transporter), the transfer (e.g., transferrin and serum ferritin), and the redistribution (e.g., transferrin receptor, ferritin) of iron under strict control on both systemic and cellular levels [24]. Most of the body iron present in red blood cells associated with hemoglobin is in a form of heme, therefore metabolism of heme via the action of heme oxygenase-1 (HO-1) is also an integral part of systemic iron metabolism [25]. The second largest pool of iron is stored within ferritin is mostly in hepatocytes and macrophages. When necessary, iron is liberated from ferritin via ferritinophagy and efficiently recycled [26]. Systematic regulation of iron homeostasis relies on the interactions between hepcidin and the iron exporter ferroportin, whereas cellular regulation of iron metabolism is carried out through the actions of iron-regulatory proteins and iron-responsive elements in a post-transcriptional way [27]. Determination of iron status relies on the measurement of serum indicators, mostly transferrin saturation, soluble transferrin receptor and serum ferritin. Additionally, protoporphyrin content of red blood cells also reflects iron status. Because of other influencing factors such as inflammation, determination of iron status is often based on the combination of several indicators [28]. 

Genetic or acquired perturbations in iron metabolism can lead to excessive accumulation of iron in the body. For example, hemochromatosis, characterized by increased dietary iron uptake and subsequent tissue iron overload, is caused by mutations in diverse genes involved in iron metabolism. In Caucasian population the most frequent type of hereditary hemochromatosis (HH) is due to a mutation of the *HFE* gene (Cys282Tyr) [29,30]. Mutations of transferrin receptor-2 gene (*TfR2*) [31], hemojuvelin gene (*HJV*) [32], hepcidin gene (*HAMP*) [33] and the ferroportin gene (*SCL40A1*) [34] contribute to less frequent forms of hereditary hemochromatosis.

Red blood cell lysis is a common feature of inherited and acquired anemias such as thalassemias, sickle cell disease and acquired refractory anemias. Massive intravascular hemolysis leads to the release of large amounts of hemoglobin and subsequent deposition of iron and hemosiderosis in diverse organs including heart, liver and kidney [35,36]. Allogeneic blood transfusion is frequently used as the first therapeutic option for the treatment of inherited anemias which improves dramatically the prognosis of these diseases, but repeated transfusions often cause secondary iron overload due to the lack of active mechanisms to remove excess iron [24,37].

Hepcidin, the peptide hormone that regulates systemic iron metabolism is produced by the liver therefore chronic liver diseases are frequently associated with iron overload. In line of this notion, high iron levels are present in patients with nonalcoholic fatty liver disease, alcoholic liver disease as well as hepatitis C viral infection [38].

Iron overload has been recognized as a potential hazard in postmenopausal women as well. The New York University Women’s Health Study showed a more than two-fold increase in the mean serum ferritin concentration in postmenopausal women as compared to premenopausal women [39]. Studies showed positive correlation between serum ferritin levels and age, menopausal status, body mass index, cigarette smoking and non-white ethnicity [39,40]. Among dietary factors serum ferritin concentrations were associated positively with intake of meat, the use of multivitamins and alcohol consumption [39,41]. Another study found that dietary habits characterized by low intake of eggs and dairy products, high alcohol consumption, and increased intake of meat rich in heme iron is associated with increased serum ferritin levels in postmenopausal women [42].

### 3.2. Bone Phenotype in Association with Iron Overload

**Figure 1:**
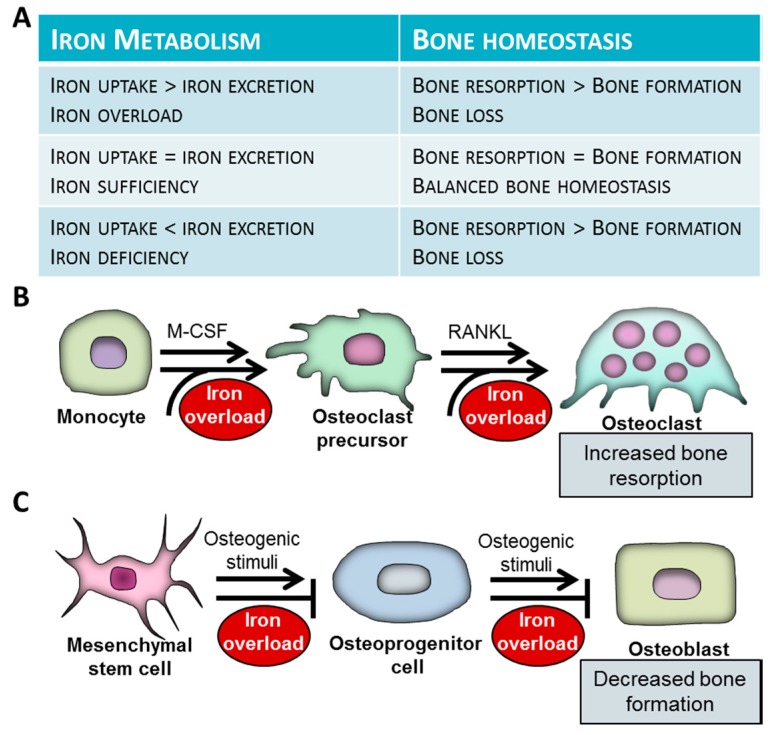
Associations of iron metabolism and bone homeostasis. (**A**) Association between iron metabolism and bone homeostasis. (**B**) The effect of iron overload on differentiation and function of osteoclasts. Osteoclasts derive from myeloid cells of the monocyte/macrophage lineage. Osteoclastogenesis is initiated by macrophage colony-stimulating factor (M-CSF). Bone-resorbing multinuclear osteoclasts are formed from mononuclear osteoclast precursors via fusion. The process is initiated by receptor activator of nuclear factor κB ligand (RANKL). Iron excess triggers osteoclast differentiation and activation and subsequent bone destruction. (**C**) Osteoblasts differentiate from multipotent mesenchymal stem cells (MSCs). Iron attenuates osteogenic differentiation of MSCs and function of mature osteoblasts. Weak bone phenotype observed in patients with systemic iron overload is a consequence of increased bone resorption by osteoclasts and decreased bone formation by osteoblasts.

Accumulating evidence suggest that iron overload conditions (HH, thalassemias, sickle cell disease) are associated with bone weakening, which is represented as decreased bone mass, osteoporosis osteopenia, altered bone microarchitecture and biomechanics, as well as frequent bone fractures [43] (Figure 1A).

There are several case reports and studies on small groups suggesting an association between HH and osteoporosis [44,45,46,47]. Based on these studies a positive correlation has been proposed between the development of osteoporosis and the severity of iron overload [44,45,46,47]. The incidence of osteoporosis in HH patients is about 25–34% and 40–80% of the patients suffer from osteopenia [46,48]. The largest case-control study of the field, with the involvement of about 600 subjects (HH patients and controls), confirmed the association between HH and increased prevalence of osteoporosis [49]. Osteoporosis can lead to pathologic fractures which have been reported in HH patients [50,51]. These studies also highlighted that the prevalence of fractures in HH patients is associated with the severity of iron overload [50,51].

Different mutations in the genes encoding the alpha and beta chains of hemoglobin (Hb) cause hemoglobinopathies which are characterized by various degrees of anemia [52]. The most common types of hemoglobinopathies are thalassemias and sickle cell disease (SCD). In case of severe or persistent anemia, patients with hemoglobinopathies may receive blood transfusion therapy which cause iron overload [52]. To avoid iron accumulation patients receive iron chelation therapy [53]. Unfortunately, regardless of iron chelation therapy, most patients with thalassemia suffer from iron overload, which aggravates the development of end-organ damage associated with the disease.

Severe osteoporosis and pathologic fractures continued to be one of the most common co-morbidity in patients with thalassemia [54,55,56]. Improvement of chelation therapy resulted that the incidence of osteoporotic fractures decreased in thalassemia patients in recent decades [57,58,59,60,61]. On the other hand, due to efficient chelation regimen life expectancy of patients with thalassemia major and intermedia increased which was unfortunately associated with increased prevalence of lifetime fractures compared to control subjects [62]. Importantly, the risk of pathologic fractures correlates with the severity of anemia and the regularity of blood transfusion in patients with thalassemia. Regardless of optimal iron chelation, regimen bone mineral density (BMD) of thalassemia patients decreases gradually by age, which leads to low BMD in more than half of the adult patients [62]. Besides bone quantity, thalassemia has a detrimental effect on bone quality. In line with this notion, it was found that trabecular bone score is decreased in thalassemia patients in comparison with healthy subjects [63].

Besides thalassemias, bone involvement is very frequent in SCD. More than 70% of adult SCD patients have low BMD that can lead to osteoporetic fractures and vertebral collapse [64,65,66,67,68,69]. According to a recent study, low BMD in SCD is accompanied with a high rate of erythrocyte lysis [70]. Iron status of SCD patients is not evident, iron overload as well as iron deficiency have been described [71,72]. This controversy may be due to the huge individual differences in iron depositions in patients with SCD and the abnormal distribution of iron that results in accumulation of iron in diverse organs including the liver, spleen and the kidney, and concomitant iron deficiency in the bone marrow [73]. Nevertheless, recently it has been shown that more than 70% of SCD patients with high serum iron suffer from low BMD, suggesting a detrimental effect of high iron on bone homeostasis in SCD [74].

Menopause is a complex process, characterized by hormonal alterations, such as a marked decline of estrogen level. Interestingly, studies revealed a negative correlation between estrogen and serum ferritin levels, and mounting evidence suggests that the 2–3-fold increase in iron/ferritin levels in postmenopausal women influences their health [75,76,77]. About one-third of post-menopausal women suffer from osteoporosis and subsequent osteoporotic fractures [78,79,80]. Recent studies addressed whether increased iron stores effect bone health in women following menopause. They showed that the rate of annual bone loss correlates to plasma ferritin levels highlighting that elevated total body iron stores is an independent risk factor for enhanced bone loss in postmenopausal women [81,82].

Interestingly, a recent study revealed that the prevalence of low BMD is lower in elderly (>60 years) people with high serum ferritin levels (<200 ng/mL). This finding warrants the need of further studies to clarify the association between serum ferritin concentrations and BMD in different populations [83].

Animal models of iron overload provided further evidence regarding the harmful effect of iron on bone health. Chronic administration of iron dextran to mice results in tissue iron overload in different organs and osteoporosis [84]. Bone loss observed in this iron overload model was linked to elevation of the production of ROS [84]. Inhibition of unfettered ROS formation partially prevented bone loss in this model, emphasizing the key role of ROS in iron-overload associated bone loss [84]. Recently it has been shown that high iron stress induces rapid osteoporosis in zebrafish larvae and adults [85]. Investigations carried out on hemochromatosis animal models (i.e., Hfe and hepcidin deficient mice and hepcidin deficient zebrafish) also supported the detrimental effect of iron overload on bone health [86,87,88,89,90]. Besides altered iron metabolism, abnormal distribution of heme seems to influence skeletal health. In line of this notion, it was shown that the mice deficient of the cytoplasmic heme exporter, feline leukemia virus, subgroup C, receptor 1 (FLVCR1), exhibit craniofacial and limb deformities [91,92]. Furthermore, several studies were performed to investigate the bone phenotype in thalassemia and sickle cell disease mice. These studies revealed that thalassemia mice exhibit the same weak bone phenotype as the thalassemia patients [93,94,95]. Similarly, sickle cell disease mice are characterized by low BMD and harmful alterations in the microarchitecture and mechanics of bones [96,97].

Additionally, chronic liver disease patients frequently suffer from osteoporosis [98,99]. The mechanism underlying chronic liver disease-associated osteoporosis is not entirely understood, but studies suggested that low BMD is mainly due to decreased bone production in these patients. Liver disease-associated osteoporosis seems to be multifactorial; retained substances such as bile acids and bilirubin, elevated iron and pro-inflammatory cytokines are assumed to play a pathophysiological role in low osteoblast activity [98,100].

Ovariectomy-induced menopause mice model was used to address whether elevation of body iron stores play a pathophysiological role in postmenopausal osteoporosis [101]. These studies revealed that excess iron does contribute to bone loss after menopause, through the generation of oxidative stress [101,102].

## 4. Bone Homeostasis in Iron Deficiency

Iron deficiency is a common disease which affects almost 1.2 billion people worldwide [103]. The most frequent consequence of iron insufficiency is anemia, due to lack of iron for heme biosynthesis. Besides its fundamental role in oxygen delivery, iron is involved in diverse enzymatic systems throughout the body. Regarding bone physiology, iron is critically involved in 2 processes, i.e., collagen production and metabolism of vitamin D and therefore iron deficiency is considered to have a detrimental impact on bone homeostasis [104,105] (Figure 1A).

Bone tissue is rich in collagen type I, the synthesis of which involves hydroxylation of pro-collagen on proline and lysine residues. The reactions of hydroxylation are catalyzed by prolyl-4-hydroxylase and lysyl-hydroxylase, the enzymes of which require ferrous iron for their catalytic activities [106,107].

Active vitamin D plays an important role in bone homeostasis through regulating intestinal uptake and tubular reabsorption of calcium and phosphate, the major inorganic components of bones [108]. Vitamin D activation is regulated by enzymes of the cytochrome P450 family which enzymes contain heme as a prosthetic groups, therefore their activities are dependent on availability of iron [109].

The effect of iron deficiency on bone health was addressed in several animal studies [104]. These studies revealed that severe nutritional iron restriction causes unbalanced bone turnover, leading eventually to bone weakening, characterized by low BMD and decreased bone mineral content [110,111,112]. Besides iron, nutritional restriction of other trace elements such as copper and selenium have been shown to compromise skeletal health [113].

On the other hand, we mostly lack information about the relevance of iron deficiency on bone health in humans. In a small study with the involvement of about 100 young iron-deficient but otherwise healthy women, the authors examined the relationship between bone metabolism and iron status [114]. This study revealed that the aminoterminal telopeptide of collagen I, that is a biomarker of bone resorption, negatively correlates to ferritin levels and concluded that iron deficiency is linked to higher rate of bone resorption [114]. Pharmacological iron treatment reduced accelerated bone remodeling in pre-menopausal women with iron-deficiency anemia [115].

## 5. Cellular Mechanisms Underlying Bone Loss in Iron-Overload

### 5.1. Iron Overload and Bone Resorption

Bone destruction is carried out by osteoclasts, the highly specialized cells originating from myeloid cells of the monocyte/macrophage lineage. Mounting evidence show that excess iron facilitates osteoclastogenesis and increases bone-resorbing activity of mature osteoclasts (Figure 1B).

As it was previously discussed, the RANKL/OPG system is the central regulator of osteoclast differentiation and activation [116]. It has been shown that iron promotes RANKL-induced osteoclast differentiation of RAW264.7 cells, as well as bone marrow-derived macrophages [117]. Differentiation of osteoclasts is associated with remarkable changes of cellular iron homeostasis promoting iron uptake, utilization and reduced iron excretion. In line of this notion, it has been shown that expressions of divalent metal transporter 1 (DMT1) and transferrin receptor 1 (Tfr1) are responsible for cellular uptake of non-transferrin bound (NTB) and that transferrin-bound iron respectively are increased, together with Steap4, an endosomal ferrireductase, that plays a key role in cellular iron utilization, while the expression of ferroportin (FPN), the only known iron exporter, is downregulated at the initial stages of osteoclast differentiation [118,119,120,121,122]. Additionally, a recent study showed that deletion of FPN in myeloid cells in mice triggers accumulation of iron and stimulates osteoclastogenic differentiation in vitro as well as in vivo [123]. Deficiency of the heme degrading enzyme HO-1 paradoxically results in accumulation of non heme iron in diverse cells in both humans and mice [124,125]. Interestingly, it has been shown that HO-1 upregulation by heme inhibits osteoclastogenesis leading to decreased bone resorption [126]. Further investigation of this phenomenon revealed that HO-1 is involved in the early stage of osteoclast differentiation induced by RANKL [127].

Osteoclasts are considered high energy demand cells as they actively pump out protons to the absorption cavity to dissolve hydroxyapatite mineral, secrete proteolytic enzymes to degrade collagen, meanwhile they need to maintain their motility [119,128,129]. This high energy demand of osteoclasts requires much mitochondria [119,128,129]. Mitochondrial biogenesis is a process highly dependent on the availability of iron, which could explain the high iron demand, and metabolic adaptation towards increased iron uptake and reduced iron excretion of differentiating osteoclasts [119].

Iron participates in ROS generation, and some studies revealed that iron-induced accelerated Production of ROS plays a central role in iron-mediated promotion of osteoclastogenesis [101,121]. A recent study showed that iron stimulates osteoclastogenesis of bone marrow-derived macrophages in a mechanism dependent on ROS production and the activation of NF-κB signaling pathway [130]. Suppression of NF-κB signaling attenuates osteoclast differentiation [130].

Growing evidence suggests that iron not only affects osteoclastogenesis, but influences mature osteoclast activity and bone resorption too. Mature osteoclasts highly express TRAP, an enzyme that catalyzes the dephosphorylation of bone matrix proteins including bone sialoprotein and osteopontin [131]. Inhibition of TRAP activity in osteoclasts abolishes bone resorption and TRAP deficient mice have a mild osteopetrotic phenotype characterized by increased amount of bone tissue and elevated mineral density [132,133]. TRAP is an iron-containing enzyme, the activity of which is dependent on ferric iron [134,135]. Expression of TRAP is regulated by iron through an iron regulatory element located at the TRAP 5’-flanking region [136].

Several in vivo studies concluded that bone-loss observed in iron-overload conditions is due to accelerated bone destruction rather than decreased bone formation. Different mice models of iron overload revealed that the osteoporotic phenotype is accompanied by elevation of osteoclast number in the bone tissue [84,88]. Furthermore, the level of C-telopeptide of type-I collagen, a serum marker of bone resorption was found to be elevated in hepcidin deficient mice, suggesting that elevated activity of osteoclasts induces bone-loss in association with iron-overload [87].

### 5.2. Iron Overload and Bone Formation

Osteoblasts derive from multipotent mesenchymal stem cells (MSCs), under the control of the master osteogenic transcription factor Runx2. Recently it has been shown that excess iron inhibits osteogenic differentiation of MSCs through the downregulation of Runx2 [137] (Figure 1C). The inhibitory effect of iron was dependent on the upregulation of ferritin, the key intracellular iron storage protein [137]. Some other studies reported that superparamagnetic iron oxide nanoparticles also impair osteogenesis of human MSCs, the effect of which is reversed by the iron chelator desferrioxamine (DFO) [138,139]. Iron loaded fetal rat calvaria cells lose the capacity to form mineralized bone nodules and show decreased expressions of osteoblast phenotypic markers, suggesting that iron not only attenuates osteogenic differentiation of MSCs, but disturbs mineralization of the extracellular matrix of osteoblasts [140].

This phenomenon was further investigated and early studies showed that excess iron attenuates proliferation as well as function of osteosarcoma cells [141]. Osteoblasts respond to iron overload by fast and persistent down-regulation of transferrin receptor and up-regulation of ferritin light and heavy chains (FtL and FtH) [140]. Parallel with these responses, suppression of osteoblast phenotype gene markers occurs, eventually leading to a reduction in the number of mineralized nodules [140]. Recently it has been shown that excess iron downregulates the expression of Runx2 and its downstream targets OCN and ALP in human osteoblasts, leading eventually to attenuation of extracellular matrix mineralization in osteoblasts [142]. Increased expression of ferritin, and in particular, ferroxidase activity of the FtH subunit, seems to play a critical role in the iron-mediated suppression of osteoblast activity and the diminished extracellular matrix mineralization [142,143]. Further studies revealed that excess iron inhibits extracellular matrix mineralization of BMP2-induced osteoblasts through a mechanism dependent on HedgeHog signaling [144,145]. Additionally high iron inhibits extracellular matrix mineralization triggered by a mixture of activated vitamin D_3_ and β-glycerophosphate via the upregulation of FtH [146].

In vivo studies revealed that decreased bone formation contributes to bone-loss in iron-overload conditions such as in th3 thalassemia mice and hemizygous β-globin knockout mice [93,95]. Additionally, Hfe deficiency is found to be associated with decreased numbers of active osteoblasts [89]. Decreased mRNA levels of osteoblast-specific proteins such as ALP, Runx2, osterix and OCN were detected in the tibia of Townes transgenic sickle mice [97]. Hepcidin deficiency was associated with decreased osteoblast activity characterized by low serum OCN level in mice and reduced mRNA levels of Runx2 and osterix in zebrafish [90]. A recent study revealed that iron treatment induces a reduction in Runx2 mRNA level in compact-bone resident osteoprogenitor cells in mice, suggesting that iron negatively influences osteogenic commitment and differentiation, in vivo [137].

## 6. Effect of Iron Deficiency on Osteoclast and Osteoblast Differentiation and Function

As discussed before, iron overload undoubtedly increases osteoclast differentiation and activity, while inhibiting osteoblast differentiation and function. The effect of iron deficiency on these cells has not been fully addressed and remained somehow controversial.

### 6.1. Iron Deficiency and Bone Resorption

Several studies suggested that iron deficiency decreases differentiation as well as activity of osteoclasts, which eventually leads to improvement of bone density. In line of this notion, it has been shown that lactoferrin, an endogenous iron-binding glycoprotein inhibits osteoclastogenic differentiation of monocytes, reduces the expression of RANKL, and improves bone density via decreasing RANKL/OPG ratio [147,148]. Further in vitro studies showed that the iron chelators clinoquinol and deferoxamine (DFO) inhibit osteoclast differentiation, as demonstrated by the attenuation of osteoclasts formation and suppression of osteoclast specific genes expression [149,150]. A recent in vivo study investigated the effect of iron chelators on the remodeling of bioceramic bone graft. The study revealed that local administration of iron chelators reduced graft resorption in correlation with a marked decrease in the number of osteoclasts at the interface of bone and the graft [151].

Ferric ion is needed for the activity of TRAP, therefore TRAP activity and osteoclast function is inhibited by a ferric chelator [134,135].

On the other hand, hypoxia response also contributes to the overall effect of iron chelation on osteoclast activity and bone resorption. Hypoxia response is regulated by the hypoxia inducible factor (HIF), a heterodimer transcription factor composed by an inducible alpha subunit (HIF-1α) and a constitutively expressed beta subunit (HIF-β). Under normoxic condition, HIF-1α is hydroxylated by prolyl-4-hydroxylase enzymes (PHDs) and degraded in proteosome. Reduced PHD enzyme activity leads to HIF-1α accumulation, nuclear translocation, dimerization with HIF-β, and binding to the hypoxia-response element of genes under the transcriptional control of HIF [152]. Catalytic activities of the PHDs require iron, and therefore, iron chelators inhibit hydroxylation of HIF-1α and activate HIF signaling [153]. It has been shown that HIF activation enhances bone resorption activity of osteoclasts [154,155,156]. This mechanism might contribute to bone loss associated with chronic iron-deficiency [104].

### 6.2. Iron Deficiency and Bone Formation

Although the negative influence of excess iron on bone formation is quite clear, we have conflicting observations about how iron deficiency affects this process. Some studies describe a negative effect of bone restriction on osteoblast function and bone formation in rats [157,158]. Other studies found that iron chelation promotes osteogenic differentiation and osteoblast activity [142,159,160]. This discrepancy might be due to the different chelation procedures used in the different studies. A recent detailed study revealed that the effect of low iron is biphasic; mild low iron increases osteoblast activity, whereas very low levels of iron inhibits osteoblast activity [161]. Iron deficiency is often associated with anemia. The effect of this condition on bone homeostasis was investigated in a zebrafish model. Iron deficiency anemia was associated with defects in bone formation, assessed by reduced number of calcified vertebrae and decreased expression of osteoblast specific genes [162].

## 7. Targeting Iron as a Therapeutic Approach to Treat Bone Loss in Association with Iron Ovreload

Because of the close association between iron overload and osteoporosis, different therapeutic approaches to decrease iron level may have clinical potential for the prevention and/or treatment of iron overload-associated osteoporosis. In clinical practice, systemic iron overload is treated with iron chelators such as DFO, deferiprone, and deferasirox [163,164,165]. The effect of the iron chelation therapy on bone health was addressed in several studies performed with the involvement of iron-overload patients. Well-designed chelation therapy has been proved to prevent the occurrence of osteopenia and/or osteoporosis in the first twenty years in in thalassemia patients [166]. Deferasirox therapy prevented bone loss and decreased the prevalence of lumbar spine osteoporosis in adult β-thalassemia major patients [167]. In a recent study, the efficiency of the different iron chelators were compared in the prevention of bone disease in β-thalassemia major patients [168]. The authors found that deferasirox significantly increased the mean BMD T-score and decreased the occurrence of osteoporosis, but DFO or deferiprone alone or in combination, had no beneficial effects on the bones [168]. Despite the fact that these studies suggested that properly designed iron chelation therapy is able to prevent iron overload-associated bone abnormalities, bone disease remained an unsolved and caused quite frequent complication in patients with iron overload [169]. This warrants further studies to optimize the chelation regimen for different iron-overload conditions, and the search for alternative therapeutic strategies for lowering iron. Hepcidin, the master regulator of iron homeostasis seems to be a promising target in the treatment of iron overload-associated bone loss [170].

## 8. Concluding Remarks

There is an evident detrimental effect of iron excess or bone homeostasis which can manifest itself in different ways, including low BMD, osteoporosis or osteopenia as well as altered microarchitecture and biomechanics. These conditions increase the incidence of pathologic fractures in patients suffering from diverse types of iron overload. The effect of iron deficiency on bone health is less clear, but some studies suggest that this condition is also associated with weakened bones, highlighting that balanced bone homeostasis requires optimal—not too low, not too high—iron levels.

Bone homeostasis involves bone destruction driven by osteoclasts, and bone formation by osteoblasts, the processes of which are interconnected and tightly regulated, assuring the maintenance of skeletal health. Differentiation as well as cellular activity of both osteoclasts and osteoblasts is influenced by excess iron resulting to a net effect on bone loss.

Osteocytes, the third cell type in bone tissue have an emerging role in bone homeostasis and remodeling, but we lack complete information about whether iron excess or deficiency influences their activity and function [171]. Additionally, further studies are needed to identify the signaling mechanisms underneath the effect of iron excess and deficiency on the differentiation and function of osteoclasts and osteoblasts.

Iron lowering therapeutic interventions could prevent or improve iron overload-associated bone abnormalities. We need further studies to reveal that keeping serum iron concentration in the target zone with the use of iron chelators can normalize bone homeostasis in patients with different forms of iron overload. Additionally, trials are needed to investigate the efficiency of iron chelation therapy in the treatment of postmenopausal osteoporosis.
